# Stakeholder participation in sustainability assessment of non-wicked problems: The case of a future seaweed industry in Sweden

**DOI:** 10.1007/s13280-021-01609-8

**Published:** 2021-10-01

**Authors:** José Potting, Jean-Baptiste E. Thomas, Fredrik Gröndahl

**Affiliations:** Present Address: Water and Environmental Engineering, Department of Sustainable Development, Environmental Science and Engineering (SEED), KTH Royal Institute of Technology, Teknikringen 10b, 10044 Stockholm, Sweden

**Keywords:** Seaweed cultivation and processing, Stakeholder interaction, Sustainability assessment

## Abstract

Acceptance by, and cooperation with relevant stakeholders in developing new sustainability initiatives when they are generally perceived as positive, is one of the keys for successful implementation of such new sustainability initiatives later on. It is remarkable, however, that ample literature exists about involving stakeholders in research projects focusing on problems with diverging views (controversy) around facts and values (wicked problems), but there is very little literature addressing whether and how to involve relevant stakeholders in case of initiatives where diverging norms and values do not play a (substantial) role, like in sustainability assessment for a future seaweed industry. This perspectives paper addresses that gap, and explores how to design such sustainability assessment, illustrated by how stakeholder interaction influenced the assessment and its results for a future seaweed industry in Sweden, followed by a discussion whether and how a similar approach may benefit sustainability assessment of other non-wicked sustainability initiatives.

## Introduction

Momentum is gathering along the Atlantic coast of Europe to capitalize on the potential of seaweed farming as a multi-value, environmentally friendly and renewable biomass. Broad interest in seaweed farming and processing was stimulated by two communications of the European Commission (EC 2012a, b). The first EC communication, seeking to accelerate economic recovery after the 2008 financial crisis, issued strategic innovation and sustainable growth of economic activities based in renewable biomass from agriculture and aquaculture (EC 2012a). The second EC communication highlighted the potential of blue growth, i.e. the marine side of the bioeconomy (EC 2012b).

**Fig. 1:**
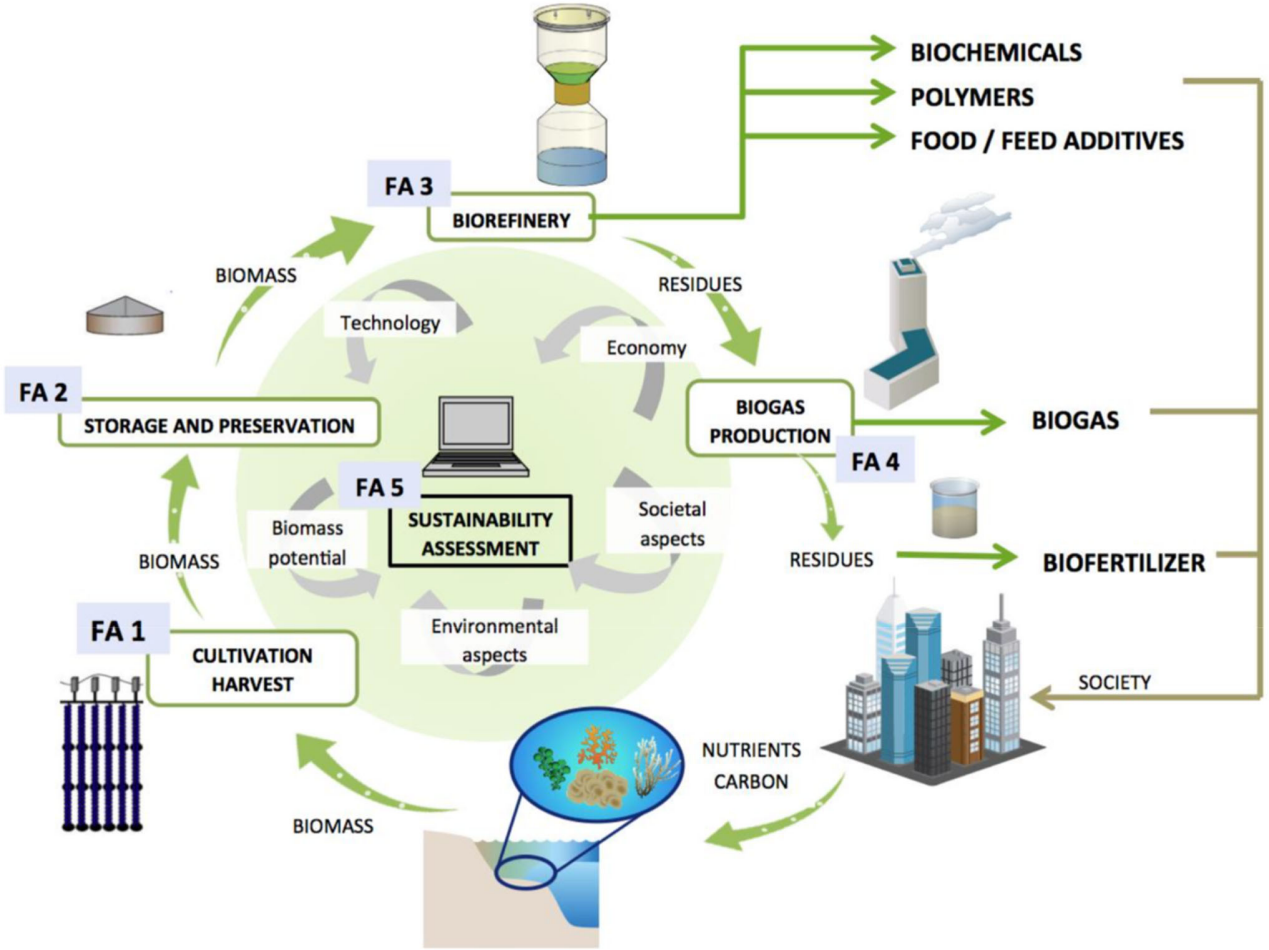
The five focus areas (FAs) of the Seafarm project [copied from project-application by Gröndahl et al. (unpubl.)]

Seaweed’s potential contribution to the economy has long been recognized in Sweden (Ackefors 1980; Edler et al. 1980; Ackefors et al. 1982; Eilola and Stigebrandt 1999; Harlén and Zackrisson 2001; Jöborn et al. 2001; Pihl 2001). In response to abovementioned EC-communications, Swedish seaweed research activities intensified and originally came together in the Seafarm project. Drawing on key expertise from five Swedish Universities, the Seafarm project aimed to lay the foundations for a future seaweed industry consisting of seaweed farming (seeding, cultivation and harvesting) and processing (preservation techniques, biorefinery processes and biogas production). A sustainability assessment was at the core of the Seafarm project (see Fig. 1).

According to the Swedish sustainable development strategy, ‘All policy decisions must take account of the longer-term economic, social and environmental implications’. Securing sustainable livelihoods and the promotion of more sustainable production and consumption of goods are thus seen as high priorities in Swedish policy making (Swedish Government Communication 2003). Also Swedish funding bodies recognize the importance of stakeholder engagement for successful development and implementation of sustainability initiatives (Formas 2019). Against this background, the Swedish research council Formas, as the financing body for the Seafarm project, posed involvement of stakeholders as an important requirement to its sustainability assessment, albeit without specifying when and how to do so.

There is a large body of literature describing or reviewing indicator frameworks covering different aspects of sustainability (e.g. Finnveden and Moberg 2005; Hak et al. 2012; Singh et al. 2012; Joung et al. 2013; FAO 2014; Sala et al. 2015; De Olde et al. 2016). Some of these references cover aspects of an assessment procedure, but none of them explicitly addresses how to involve relevant stakeholders.

There is ample literature suggesting that sustainability assessments are most effective when all relevant stakeholders accept its process and results as credible (they perceive scientific methods and results as robust), salient (assessed issues matter to them) and legitimate (they feel fairly represented in the assessment process). This literature typically focuses on so-called 'wicked problems' in which borders between facts and values are fading and contested, and where stakeholder participation is put forward to adequately deal with those controversies (Eckley 2001; Cash et al. 2003a; Tippett et al. 2007; Hage et al. 2008; Reed 2008).

Another interesting body of literature focuses on the merits and ways of involving stakeholders in sustainability assessments in the area of biodiversity and nature conservation particularly as to unlock tacit knowledge intertwined with tacit views that are only accessible through these stakeholders. Views and tacit knowledge of these stakeholders do not necessarily clash with but may diverge from scientific knowledge of and views about given problems. This body of literature is directed at bridging scientific knowledge and views with local non-scientific (indigenous) knowledge and views through a process of knowledge sharing between scientists and stakeholders to produce useable new forms of knowledge (Tengö et al. 2017; Folkert et al. 2020).

In 2013, at the start of the Seafarm project, there was little controversy around a future seaweed industry. Its development was widely assumed to be a win–win initiative, positive for both the economy and the environment. Unlocking-related tacit knowledge of local communities was neither seen as a barrier to future developments. The main issue in that early stage of exploring a future seaweed industry, when the Seafarm project started and prior in the application process for project funding, was rather a lack of scientific knowledge about technical, economical and sustainability aspects of such future seaweed industry.

There seems little literature about stakeholder participation in sustainability assessment of such non-controversial or non-wicked problems where a lack of scientific knowledge is the core issue. To that purpose, this paper brings together pieces of literature about stakeholder participation in sustainability assessments, illustrates how the stakeholder participation influenced the assessment and its results in the Seafarm project and discusses whether and how in our opinion a similar approach may benefit sustainability assessment of other non-wicked problems, before drawing conclusions.

## Theoretical background

### Problem archetypes

**Fig. 2:**
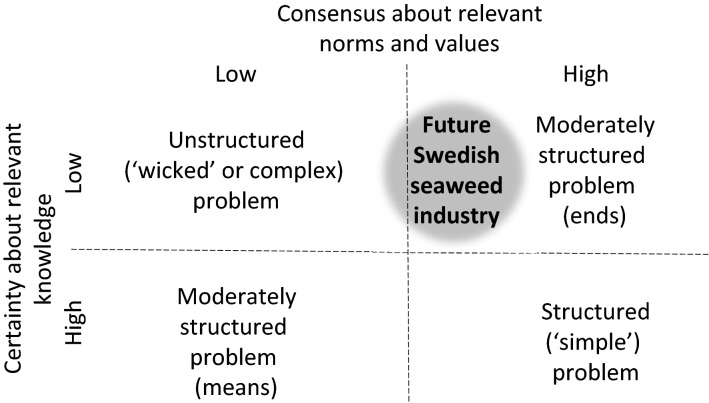
Quadrant of four archetypes of policy problems in which the Seafarm project positioned in the right up corner. (adapted from Hisschemöller and Hoppe 1996)

We are of the opinion, similar as Hurlbert and Gupta (2015) that whether and how to involve stakeholders in a sustainability assessment depends on the nature of the problem considered. Hisschemöller and Hoppes (1996) distinguish between four archetypes of societal problems in a quadrant that plots consensus about relevant norms and values on one axis and certainty about relevant knowledge on the other (see Fig. 2).

Structured or simple problems are those for which there is consensus around relevant norms and values as well as certainty about relevant knowledge. That is, stakeholders agree on how they define the problem, and the existing body of knowledge allows solving it. Unstructured, wicked or complex problems are usually understood as those where stakeholders (strongly) disagree about whether and how they see a problem and thus also about solution directions, whereas also (strong) disagreement on the robustness of involved knowledge is part of the controversy (Termeer et al. 2019). Climate change is often put forward as the ultimate example of an unstructured (wicked) problem in which both norm and values as well as the certainty of the knowledge were and still are contested (Björnberg et al. 2017).

Wicked (unstructured) problems are typically viewed as surrounded with controversy around facts, norms and values. Controversy about norms and values was not a core condition though for wickedness according to Rittel and Webber (1973), who coined the term ‘wicked problems’, but rather a consequence of a problem-solving attitude in which professionals (here researchers) assumed their problem diagnoses were of a generic nature and therefore shared by the public. Such problem-solving attitude, following a classical paradigm of science and engineering, may work for definable and separable problems (i.e. structured or simple problems in Fig. 2). It does not typically work for wicked problems defined by the process of formulating the problem and of conceiving a solution being identical (Rittel and Webber 1973; Termeer et al. 2019). This also encompasses problems without controversy about facts, norms and values, but that in exploring causes and solution nevertheless require unlocking tacit knowledge exclusively accessible through local (indigenous) stakeholders (Tengö et al. 2017; Folkert et al. 2020). Thus, we consider this type of problem as well as unstructured (wicked) problems, based on the similar challenge of bringing together diverging views on facts, norms and values in the assessment process.

The two archetypes of moderately structured problems fall in between structured and unstructured problems. In case of the one archetype, stakeholders agree on the problem definitions, but lacking or uncertain knowledge (means) prevents solving a moderately structured problem. The opposite applies to the other archetype of moderately structured problems. Sufficient knowledge is available, and stakeholders do not dispute its robustness but deviate in how they evaluate the given problem and solution directions (ends). Thus, either certainty about knowledge lacks, or consensus about norms and values is missing for moderately structured problems.

### Types of stakeholder participation

Reed (2008) discusses four (related and overlapping) typologies of stakeholder participation (the nature of problems is not included as a typology). Table 1 combines the four typologies of Reed (2008) with their implementation by Hage et al. (2008). For the first typology, the degree of interaction (1st column in Table 1), we prefer the ‘participation ladder’ of Hage et al. (2008) as more neutral than the widely used version of Arnstein (1969) with normatively phrased rung names implying that higher levels are always better than lower levels of interaction (Seidl 2015). Reed’s (2008) second typology is about the direction of the information flows (2nd column in Table 1). In parallel with the degree of interaction, the information flow runs from two directional at the higher rungs, through one directional at the middle rungs, to non-existing at the lower rungs of the participation ladder (Hage et al. 2008). The third and fourth typologies of Reed (2008) are in Table 1 taken together by assigning the four reasons for stakeholder participation (i.e. substantive, instrumental, equity and democracy, empowerment; 4th typology) from Hage et al. (2008) to either its normative or pragmatic basis (3rd typology).

**Table 1:**
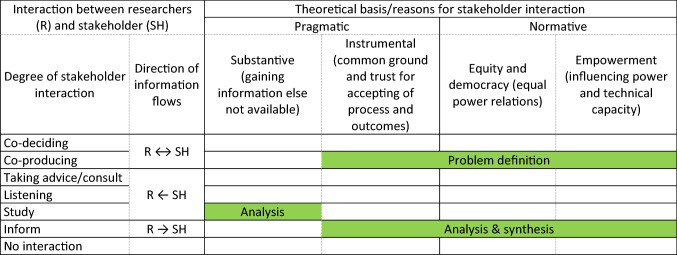
Four typologies of stakeholder participation from Reed (2008) with their implementation by Hage et al. (2008): Degree and direction of and reasons for interaction between stakeholders and researchers (Hage et al. 2008; Reed 2008), and in green indicated how this was implemented in the sustainability assessment of the non-controversial future seaweed industry in the Seafarm project

According to Hurlbert and Gupta (2015), who also refer to Hisschemöller and Hoppe’s (1996) archetype problems presented in Fig. 2, all but structured (simple) problem types require relatively high degrees of stakeholder involvement to establish and/or maintain trust of all stakeholders in the stakeholder process. Hurlbert and Gupta (2015) do not make explicit why a relative high level of stakeholder participation is needed in case of moderately structured problem with uncertain knowledge (but with no to little debate on norms and values). Millar and Wyborn (2020) provide a possible explanation in their review of the meaning of co-production across three scientific disciplines, i.e. public administration, science and technology, and sustainability science:

The term co-production emerged in the 1970s when public services were observed as inherently co-produced by public administrative bodies and citizens, e.g. through citizens helping the police by sharing crime information or through parents supporting their kids to learn at school. At about the same time, the idea took shape of scientific knowledge inherently being the product of the social context in which it is co-produced. When science is socially constructed, this then consequently would apply to the authority of science in the social debate, making scientific authority of interest to social institutions other than scientific organizations (Millar and Wyborn 2020).

The developments in public administration and science and technology came together in sustainability sciences with the explicit aspiration of being created through the processes of co-production in which scholars and stakeholders interact to define important questions, relevant evidence and convincing forms of argument. This resulted in the exploration of approaches for co-producing knowledge between scientists and users of scientific knowledge in the areas of, e.g. climate change (often about dealing with controversies around science and norms and values), as well as biodiversity and nature conservation (typically focusing on unlocking tacit knowledge and views)) (Bremer and Meisch 2017; Millar and Wyborn 2020).

According to Millar and Wyborn (2020), co-production as an *aspiration* in sustainability sciences is at odds with co-production as a defacto reality in public administration, and science and technology. However, the three disciplines share as a key-insight that knowledge and action are interdependent. Among the key lessons to be learned from the history of co-production, according to Millar and Wyborn (2020), there is that inclusion of a diversity of stakeholders with their accompanying power in a co-production process enables credibility, salience and legitimacy. Hurlbert and Gupta (2015) do not use the latter three terms. These seem instead to be covered by the concept of trust of stakeholders in an assessment that in the case of unstructured and moderately structured problems asks for high participation according to Hurlbert and Gupta (2015), or in all cases asks for co-production according to Millar and Wyborn (2020).

In our opinions, different from Millar and Wybornb (2020) and similar as Hurlbert and Gupta (2015), not every assessment necessarily needs co-production as the highest form of participation (see Table 1). We agree with Hurlbert and Gupta (2015) that structured problems need little or no interaction (trust not at stake) and that unstructured (wicked) problems and moderately structured problems with debate (mainly) about norms and values need higher degrees of (two-directional) interaction (trust about norms and values at stake). We like to add a need for higher degrees of (two-directional) interaction in case of unstructured problem where stakeholders have diverging but not necessarily conflicting views on facts, norms and values. We disagree with Hurlbert and Gupta (2015), however that moderately structured problems with uncertain knowledge always need higher degrees of stakeholder interactions in all phases of an uncertainty assessment. Trust in norms and values is not at stake there.

Instead, we follow Seidl et al. (2013), Seidl (2015) and Hisschemöller et al. (2001) in that sustainability assessment always needs a high degree of (two-directional) interaction with stakeholders in problem definition as to adequately cover their concerns (salience) and to give them fair influence (legitimacy) (Eckley 2001; Linke et al. 2011; Seidl 2015). This also applies to moderately structured problems where knowledge is the main issue. In subsequent analysis and synthesis; however, we deem such high degree of interaction a potential source for stakeholder fatigue (Reed 2008) and not a priori essential as long as controversy about norms and values remains small or absent. The latter needs consent of and regular check-up with stakeholders. We also do consider it essential to keep stakeholders informed about progress, but this can be done by (one-directional) informing them. When stakeholders are the only source of information, acquiring their knowledge might furthermore rely on a (one-directional) study, e.g. through a survey.

## Stakeholder participation in the Seafarm project

### Sustainability assessment design and stakeholder interaction strategy

Interactions with a variety of stakeholders during preparation of the application as well as at the start of the project convinced the research team for the Seafarm project, i.e. Seafarm researchers, of a broad consensus among relevant stakeholder about seaweed farming and processing as positive activities. Seaweed was broadly considered environmentally friendly and useful, but largely untapped, whereas its profitability was considered one of the main concerns for this budding industry. Some risks in terms of conflicts in norms and values were identified, for instance possible conflicts with private leisure boating in areas potentially suited to aquaculture, while aquaculture itself at that time already was a topic subject to controversy which could also transfer to future seaweed farming. The Seafarm project team did identify, however, considerable uncertainty about relevant knowledge. For instance, little robust knowledge was available from comparable case-studies about the direct influence of seaweed farming on its surrounding seawaters, or about the potential contribution of seaweed industry and processing to the regional economy. There were also potential risk factors, bottlenecks, and technicalities in the supply chain to overcome.

In summary, Seafarm researchers determined that the development of a Swedish seaweed industry was met with relatively good consensus about norms and values (with minor risks), but encountered large gaps in key knowledge areas. The problem was thus perceived as falling primarily in the upper right sector of the quadrant of Hisschemöller and Hoppe (1996), with minor overlaps to the upper left sector for unstructured (wicked) problems (based on above mentioned possible risks associated with conflicts in norms and values). Following Seidl et al. (2013), Seidl (2015) and Hisschemöller et al. (2001), this suggests that an effective strategy for the sustainability assessment would entail two-directional interaction between Seafarm researchers and stakeholders for identifying the relevant knowledge gaps as part of ‘problem definition’, but that researchers could stick to keeping stakeholders informed (one directional) about the progress of the project in the ‘analysis phase’ and ‘synthesis phase’, given the perceived non-controversy around a future seaweed industry. The overlap with the wicked problem sector implied a potential need for additional stakeholder interaction as part of the ‘analysis phase’ to monitor for signs of aforementioned conflicts in norms and values.

**Fig. 3:**
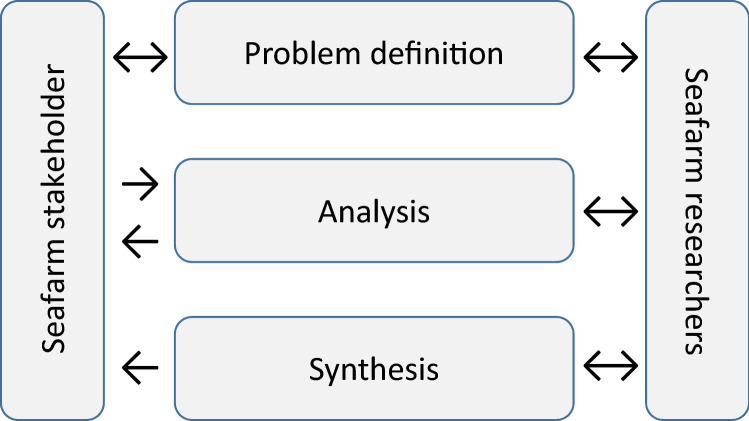
Stakeholder participation strategy for sustainability assessment in the Seafarm project (Thomas 2018)

Table 1 and Fig. 3 represent the basic strategy for stakeholder participation for the sustainability assessment in the Seafarm project. At the start of the Seafarm project, in the ‘problems definition’ phase in Fig. 3, a stakeholder workshop with two-directional interaction between stakeholders and Seafarm researchers identified currently experienced and possible future problem areas around a Swedish seaweed industry. Based on the identified problem areas, with consent of the participating stakeholders, the Seafarm project team decided on which themes to focus the subsequent ‘analysis phase’. The survey to monitor possible developments of conflicts in norms and values amongst residents of the Swedish west coast involved a one-directional information flow from stakeholders to researchers. Stakeholder interaction for the other analytical studies was also mainly one directional, albeit the other way around. Researchers did share results to stakeholders through regular communications and meetings (Thomas 2018). Some two-directional stakeholder interaction took place, for instance at project meetings during which feedback was received on ongoing research and preliminary results. Finally, an integrated overview of the sub-projects of the sustainability assessment in the ‘synthesis phase’ was planned to be presented to stakeholders in an end-of-project conference in June 2020.

This strategy for stakeholder participation was at the start of the project discussed and approved by the Swedish research council as the financing body for the Seafarm project. It was also presented to and accepted by stakeholders in the workshop at the start of the project. The regular stakeholder meetings, which served to present latest findings to stakeholders, also provided the opportunity to re-evaluate the stakeholder participation strategy in light of the project’s progress (i.e. whether or not to intensify stakeholder interaction regarding specific issues).

### Stakeholder workshop for problem identification

The stakeholder workshop for problem identification took place on April 2014. A total of 50 invitations were sent, covering a range of stakeholders, including project partners, the Swedish research council Formas, municipalities, regional government, government agencies, companies, individual researchers and research groups, and other interested groups such as Vattenbrukcentrum Väst. Vattenbrukcentrum Väst is an organization that represents a range of aquaculture-related interest groups including members of the public, local non-governmental groups, and businesses. Table 2 provides an overview of invitation response and stakeholder types, categorized by respective employments.

**Table 2:** Overview of types of stakeholder (categorized by employment) and their particular focus group interests, as well as their participation status (accepted and participated in bold before brackets; inside brackets: accepted but cancelled, could not attend, no response; bold italic indicates that some included participants have an interest in two focus areas)

Focus area (FA)	Type of stakeholder				Total
	Corporate	Researchers	Government/ local authorities	Other	
FA1 Cultivation & harvest	**1** (1, 0, ***1***)^a^	**3** (2, 0, 0)	***1*** (0, 0, 0)^b^	0 (0, 0, 0)	***5*** (3, 0, 1)
FA2 Storage & preservation	**1** (0, 0, 0)	**1** (0, 0, 0)	0 (0, 0, 0)	0 (0, 0, 0)	**2** (0, 0, 1)
FA3 Biorefinery	0 (0, 0, 1)	**2** (0, 0, 1)	0 (0, 0, 0)	0 (0, 0, 0)	**2** (0, 0, 2)
FA4 Biogas production	**1** (0, 0, 0)	**1** (0, 0, 1)	***1*** (0, 0, 0)^b^	0 (0, 0, 0)	***3*** (0, 0, 1)
FA5 Sustainability assessment	0 (0, 0, ***2***)^a^	**1** (0, 0, 2)	***2*** (0, 0, 0)^b^	0 (0, 0, 0)	***3*** (0, 0, ***4***)
General	**1** (0, 2, 4)	**2** (0, 5, 2)	**1** (2, 3, 1)	0 (0, 6, 0)	**4** (2, 16, 5)
Total	**4** (1, 2, ***7***)^a^	**10** (2, 5, 6)	***3*** (2, 3, 1)^b^	0 (0, 6, 0)	***17*** (4, 16, ***13***)

^a^One corporate invitee, who did not respond, was allocated to two FAs (namely F1&5), but was counted only once in the total

^b^Two participants from government and local authorities were allocated to two FAs (namely FA1&5 and FA4&5), but were counted only once in the total

Invitations were primarily sent out to stakeholders that actively showed professional interest or financially supported the Seafarm project. In addition, a considerable number of researchers, from Sweden and other countries, were invited for their expertise on seaweed, aquaculture or marine sciences. At that time, fewer than 100 people worked with marine aquaculture across the whole of Sweden. Of the 50 invitees, 17 showed up as participants to the workshop (i.e. a turn-out of 40% was achieved), with a higher turn-out achieved for researchers (56%) compared to the other groups combined (24%). Researchers were heavily overrepresented with 10 participants compared to 4 corporate participants and 3 participants from government and local authorities (2 of the latter covered 2 FAs). It should be noted that participants from each of these groups, notably the researchers, also represented the views or concerns of other interest groups (e.g. local non-governmental groups, leisure or environmental groups, etc.) through their involvement in aquaculture networks, environmental groups, and past or ongoing projects about a range of related issues. Furthermore, many of the participants were residents of the West Coast and, thus, could also be said to indirectly represent local communities and some degree of indigenous tacit knowledge.

The 17 invitees that accepted and participated in the workshop were split into four mixed working groups to stimulate more parallel discussions. Each working group was moderated by one of the researchers involved in FA5 (see Fig. 1 and Table 2). The moderator was also tasked with making audio recordings of the discussions within each working group as well as summarizing key discussion points on post-its and arranging them on flip-overs for a plenary presentation. The audio recordings and flip-overs were organized and condensed after the workshop into summary posters (Thomas 2014).

The workshop was organized into 3 successive brainstorming sessions. Each brainstorming session was followed by a poster presentation from each group. The aim of the first brainstorming sessions was to identify currently experienced and possible future problem areas. Each problem area was defined, discussed and documented. The second session aimed at evoking creativity and reaching consensus about a shared desirable future for the seaweed industry. In the third brainstorming session, building on the results of the previous two sessions, the working groups were tasked with suggesting ideas to overcome specific identified problem areas and pathways to their shared and desirable future.

The stakeholder workshop in April 2014 provided a range of sustainability concerns and problems, some of which were instrumental in shaping the analytical studies. The following sections present those key sustainability concerns and problems, hereafter represented by quotes from the summary posters of the workshop, and explain how they influenced subsequent analytical studies. A separate section, i.e. “Stakeholder workshop for problem identification” is devoted to the survey conducted to monitor for possible developments of conflicts in norms and values amongst residents of the Swedish west coast. The other analytical studies are covered in section Other analytical studies.

### Potential threat of public aversion

One of the sustainability concerns and problems put forward in the stakeholder workshop in April 2014 was embodied in the following quote from the summary posters (Thomas 2014, 2018):

‘Permits are likely to be a complicated obstacle for further expansion of the West coast algae industry, due to aesthetics issues and related risks of public aversion from locals and summer residents, competition with other water uses (e.g. leisure boating) and a lack of a legal framework to certify coastal aquaculture’.

The participants in the workshop identified aversion against seaweed farming as a significant risk for the development of a seaweed industry on the West Coast of Sweden. The Swedish West Coast is of great cultural and natural heritage. It has a long coastline of natural beauty and with ample opportunity for leisure boating. Leisure boating might clash and lead to conflicts with marine aquaculture, notably mussel and seaweed farming, and this may lead to difficulties in obtaining seaweed farming permits. Also, the public’s perceptions of the aquaculture sector (as a whole) could be negative as a result of the environmental problems associated with fish farming, according to the workshop participants. The workshop participants, therefore, suggested to study if and how the public might differentiate between seaweed farming, mussel farming and fish farming, notably in terms of environmental impacts and to explore their reaction and gauge the likelihood of aversion to a set scenarios for seaweed farming development on the Swedish West Coast. Such a survey of public perceptions could also be a useful benchmark of attitudes that could be revisited and compared to in the future, once these aquaculture activities might have developed to significant scales.

A web-based panel survey was conducted in 2015. The survey was randomly distributed to members of the Norstat Panel with registered addresses in the study area (Norstat is a market research company). Specific age and gender targets were set for the study area to achieve a moderate degree of sample representativity. Members were offered a small financial compensation (40 SEK to 5 $US) to complete the survey. In total 700 respondents completed the questionnaire, of which 695 provided usable answers. When asked about their general opinions on different types of aquaculture, respondents tended to be favourable though a majority chose neutral responses to most questions, indicative of a general lack of knowledge about aquaculture. Overall, respondents were favourable to the scenarios depicting future aquaculture developments on the Swedish West Coast. Finally, it was found that the high-awareness group tended to be more supportive than the low or medium-awareness groups, hinting at the benefits of increasing awareness to reduce risks of public aversion and to support a sustainable development of aquaculture on the Swedish West Coast (Thomas et al. 2017).

### Other analytical studies

“Potential threat of public aversion” section described how the work survey followed from the stakeholder workshop in April 2014 and presented the results of this survey, because residents of the Swedish west coast as stakeholders were at the centre of this survey. They were, more specifically, the subject of research for this analytical study. This section focuses on how the stakeholder workshop resulted in the other analytical studies. These studies were of a more technical character and stakeholders played no major role in them. The result of these other analytical studies, thus, says little about stakeholders and their relation with a future seaweed industry and are, therefore, not included in this section but summarized for the interested reader in Box 1.

In addition to public aversion potentially complicating permit processes, as covered by the quote in “Potential threat of public aversion” section, Seafarm researchers identified environmental performance requirements in European legislations for biofuels replacing fossil equivalents as another concern in the month following the stakeholder workshop. The European Union Renewable Energy Directive (EU 2018) requires reducing greenhouse gas (GHG) emissions for biofuels as compared to its fossil equivalent by 50–70%, depending on the application of the biofuels. The 2018 version of the directive is a recast of the 2009 version (EU 2009) requiring GHG reductions compared to a gasoline fossil fuel reference (with emissions of 83.8 g CO_2_eq/MJ) of 35% until January 2017, 50% from January 2017 and 60% from January 2018 onwards. The 2009 version of the directive was still valid around the time of the workshop (i.e. April 2014). An analytical study was performed to quantify avoided greenhouse gas (GHG) emissions and the energy return on investments (EROI) for two systems producing biogas from seaweed (specifically the brown seaweed, i.e. sugar kelp), i.e. the 0.5 ha pilot seaweed farm and biogas and fertilizer biorefinery in the Swedish Seafarm project, and a same system scaled up and adjusted to a farming area of 10 ha. Results for this study are included in Box 1 under ‘Requirements on energy performance and greenhouse gas emissions’.

The working groups also discussed a range of topics related to seaweed farming, more specifically labour and costs reducing strategies for farming infrastructure designs and seeding methods. Further, whereas the supposed environmentally friendly character of seaweed farming was considered a critical condition for its development, the working groups identified a lack of (affirmative) environmental impact studies of seaweed farming and processing. This resulted in the following quote (Thomas 2014, 2018):

‘Locally adapted and genetically diverse specimens of Saccharina Latissima will be the most productive and resilient to cultivate. Furthermore, new cultivation technologies are emerging that could reduce labor requirements, facilitate seeding, reduce environmental impacts and cut costs’.

One group of environmental impacts brought forward was direct effects on local seawater in which seaweed farming takes place (e.g. effects on benthic habitats, risks to seaweed farming due to diseases from monoculture, risks to existing local marine species from farming non-native species). At the time of processing the workshop results, it had already been determined that these questions would not be the focus of the Seafarm project, but rather that of a sister project with a focus on assessing direct local effects. That sister project would conclude that seaweed cultivation has mild and positive effects on sea bottom life and mobile plants, but few noticeable impacts were detected on oxygen fluxes and nutrient levels before and after cultivation (Visch et al. 2020).

A different type of environmental impact, however, was that over the whole seaweed farming and processing supply chain. Environmental impact studies of the seaweed supply chain, i.e. life-cycle assessments (LCAs), can shed light on questions such as whether the sequestration of carbon in the biomass is larger than related supply chain emissions, and relating to environmental supply chain optimization. A first LCA explored the environmental impacts from seaweed farming up to, and including drying the harvested seaweed by a heated air cabinet. The results of this LCA can be found in Box 1 under ‘Environmental impact of seaweed farming infrastructure and drying by heated air-cabinet’. A second more detailed LCA extended the first explorative one with two alternatives for producing seeding lines in the hatchery (spray and submersion seeding of strings with juvenile seaweed) and three additional preservation methods (hang drying, freezing and ensiling of harvested seaweed), while covering conventional single longline infrastructure as actually used in the Seafarm project. Thereby this LCA, different from the previous explorative one, explicitly related to the Seafarm project’s supply chain. The results of this LCA are presented under ‘Environmental impact of the Seafarm project’s supply chain’ in Box 1.

Several working groups furthermore discussed the feasible scale of seaweed farming and processing. Questions were raised about the space available along the Swedish west coast for seaweed farming and processing without hampering existing marine activities, space requirements to seaweed farming (e.g. depth limitations, exposure to currents, waves and storms), and related amount of seaweed that could be produced in the future. This led to the following quote (Thomas 2014, 2018):

‘The algae potential of the Swedish West coast should be estimated to assess the long-term potential and sustainability of this industry’.

The researchers found data for present and planned seawater uses on websites of some municipalities, agencies and organizations. However, no official drafts of marine spatial plans for the Skagerrak were available at the time of this study, resulting in large possible gaps in data. Therefore, a Geographic Information Systems-based Multi Criteria Decision Analysis (GIS-MCDA) study was performed to quantify the algae potential by identifying locations for seaweed farming through combining present seawater uses with location factors for seaweed farming. The results are summarized in Box 1 under ‘Seaweed potential of the Swedish West Coast’.

Economic viability was a core take home message and warning from the stakeholder workshop, given that seaweed farming can be costly notably due to labour, harvested seaweed has a relatively low value, and there is not yet a substantial and established market for high-value seaweed products (e.g. food products). This was represented in the following quote (Thomas 2014, 2018):

‘Economic viability is likely to depend on the results of FA3 research, notably the value and volume of products than can be fractioned from the algae’ and ‘The provision of ecosystem services should be understood and accounted for’.

Previous attempts for developing seaweed farms in Sweden, such as that led by Von Wachenfeldt, were unable to subsist due to a lack of profitability (Edler et al. 1980). Some key issues needing clarification, brought forward in the stakeholder workshop, were supply chain economics, cultivation costs, returns on investment, business model projections and the development of high-value products to balance the economy. In addition to these, during the workshop, it was suggested that ecosystem services, such as nutrient bioremediation, should be accounted for and monetized where possible, to provide a more holistic perspective on the economic situation. So this was what the 6th analytical study of the sustainability assessment did. The results are included in Box 1 under ‘Economic potential of a Swedish seaweed industry’.

### End-of-project conference

An end-of-project conference was planned in June 2020 to give an integrated overview of the sub-projects of the sustainability assessment. The Covid-19 pandemic regrettably necessitated indefinitely postponing this conference. Given the size of the project, Formas chose to delay the end-of-project conference until physical meetings are possible again rather than having a digital alternative (or cancel the event). It may not be until Autumn 2022 before physical meetings are possible again. At the time of submitting this article, Formas had not yet decided how to deal with this end-of-project conference.

In the absence of final stakeholder input, the Seafarm research team has reflected on the earlier fruitful stakeholder participation strategy. Areas of concerns gathered in the stakeholder workshop at the start of the project clearly guided the analytical studies undertaken during the ‘analysis phase’, including an in-depth survey of stakeholders’ attitudes towards seaweed. The survey brought potential conflict areas to light, whereas the other studies produced additional knowledge necessary to support this budding seaweed industry in Sweden. “Reflections” section gives a few critical reflections from the perspective of the Seafarm researchers.

## Reflections

Literature about stakeholder involvement typically focuses on problems for which they disagree about norms and values and often also about facts (Eckley 2001; Cash et al. 2003b; Tippett et al. 2007; Hage et al. 2008; Reed 2008). A review of process designs for stakeholder participation by Tippet et al. (2007) shows, indeed that these designs typically relate to problems with debate about norms and values (and facts). Stakeholder interaction in these process designs often exceed what the research team deemed necessary for the Seafarm project, given a positively received future seaweed industry in Sweden. Seafarm researchers perceived the quadrant of Hisschemöller and Hoppes (1996) as instructive in designing their stakeholder participation strategy. Distinguishing between certainty about knowledge on the one hand and the degree of consensus about norms and values on the other hand, were instrumental in determining the degree of interaction that would be helpful in different phases of the Seafarm project. “Stakeholder participation in the Seafarm project” section describes Seafarm’s stakeholder participation process and how it influenced the project retrospectively, in light of the theoretical background presented in “Theoretical background” section about stakeholder participation in sustainability assessments.

The stakeholder process for the Seafarm project was well thought through. It would have been beneficial to the stakeholder process, however, if more guidance about stakeholder participation in sustainability assessment for other non-wicked sustainability initiatives had been available. There are two critical reflections we would like to make about when and whom to involve in the participation process.

The development of a future Swedish seaweed industry seems to be an initiative which stakeholders hold a generally positive attitude. This consensus, however, may not be permanent. Values and perceptions can change when knowledge emerges or initiatives come closer to (large scale) implementation. Further, the general lack of awareness about aquaculture amongst residents of the west coast, as identified in the survey, can be seen as a possible threat given that a lack of awareness could lead to conflicts and aversion to future developments (Thomas et al. 2017). Thus, an initiative might evolve from a moderately structured to an unstructured problem. This was not the case during the sustainability assessment studies undertaken as part of the Seafarm project, though it may yet happen as the sector scales up in the coming years. Involving stakeholders must, therefore, remain a point of attention in spin-off activities of the Seafarm project.

Residents of the Swedish west coast and leisure or environmental interest groups were not actively involved in the Seafarm project, but rather their involvement could be considered indirect through certain stakeholders. Vattenbrukcentrum Väst, for instance, represented aquaculture-related interest groups in the stakeholder workshop. Similarly, some of the other participants represented the views or concerns of other interest groups through their involvement in environmental groups and past or ongoing projects relating to a range of related issues. Furthermore, most participants were also residents of the West Coast. The Seafarm researchers did not consider the indirect involvement of interest groups as a problem for this new sustainability initiative generally perceived as positive, but one may see this as remarkable when acceptance by and cooperation with relevant stakeholders are seen as key for success. An open question remains whether it would have made a difference for the future of the Swedish seaweed industry if these interest groups had also been directly involved in the Seafarm project.

One can argue that the stakeholder participation strategy in the Seafarm project is not fundamentally different from the ones followed in many research projects (Seidl 2015). In our experiences, these research projects often focus on problems where a lack of knowledge rather than discussion about norms and values is at stake. When they employ a stakeholder interaction process, this is sometimes in response to requirements set by respective funding bodies. As mentioned in the introduction, funding bodies increasingly recognize the importance of stakeholder engagement for successful development and implementation of sustainability initiatives (Swedish Government Communication 2003). They seldom specify when and how to do so, however, leaving researchers the task of determining a suitable strategy and how to implement it in their project.

There are as such plenty of workshops and handbooks on stakeholder participation, see e.g. the ‘Science for policy handbook’ of the European Commission (Sucha and Sienkiewicz 2020), the ‘Handbook for stakeholder engagement’ from the United Nations Environmental Program (UNEP 2020) or BiodiveERsA’s ‘Stakeholder engagement handbook’ (Durham et al. 2014). These handbooks list reasons for involving stakeholders and mention less or more participation of stakeholders may be sought. Next these handbooks, similar as the scientific literature, elaborate on higher forms of participation (notably co-production). Neither handbooks nor scientific literature, however, give clear guidance on why more or less participation is needed.

We feel that clearer guidance on a structured analysis of what level and accompanying approaches of stakeholder participation is needed, particularly also addressing problem types served by research based in a more classical paradigm of science and engineering where knowledge production is key. While we support the plea of Seidl (2015) for a more structured approach to stakeholder participation and also the plea of Reed (2011) to shift the emphasis from selecting tools to the process of stakeholder interaction as the latter strongly influences the quality of decisions made, we thus call for extending this with guidance on identifying which level and types of participation best suits the problem at stake. We encourage differentiating between processes according to the nature of problems addressed, i.e. the four archetypes of Hisschemöller and Hoppes (1996). It would also be practical, as explicitly done by Seidl et al. (2013) and Seidl (2015) and by us here as well, to account for differences in need across the phases in a sustainability assessment.

The need for transdisciplinarity [interaction between researchers and stakeholders (Seidl 2015; Walsh et al. 2021)] and interdisciplinarity [integrating interaction between disciplines (Seidl 2015; Walsh et al. 2021)] in transformative sustainability assessment has often been emphasized (Reed 2008; Gibson 2013; Seidl et al. 2013; Seidl 2015). Both need the skills and the will to relate pieces of information coming from different sources, i.e. from different scientific disciplines or from both the scientific field and from stakeholders. Walsh et al. (2021) discuss approaches, on a rather theoretical level though, for such relational thinking from its ontological, epistemological and ethical angles. Further research to develop practical approaches for such relational thinking would make a valuable contribution. In order to support interdisciplinary collaboration, higher education institutions need to remove institutional barriers to collaboration and implement policies that encourage researchers from different disciplines to work together, for examples, as co-supervisors on PhD projects.

## Conclusions

Literature about involving stakeholders in research projects typically focuses on problems with different views on facts and values (wicked problems) which can lead to controversy, but it does not usually address whether and how to involve relevant stakeholders in the case of initiatives where the main focus is on knowledge development rather than managing differing values, as in the case of a future seaweed industry. Acceptance by, and cooperation with relevant stakeholders in developing new sustainability initiatives, also when they are generally perceived as positive, is nevertheless important to avoid potential controversy later on and because it improves the quality of sustainability-related decision making, depending though on the process leading to them. Seafarm researchers considered the quadrant of Hisschemöller and Hoppes (1996) as very instructive for designing a stakeholder participation strategy for their sustainability assessment. This paper conveys the background and results as a reference and source of inspiration for other sustainability assessments, and to encourage publications with substantiated guidance for stakeholder interaction in case of non-controversial sustainability initiatives. We feel the quadrant of Hisschemöller and Hoppes (1996) serves as a powerful starting point for science to further elaborate on which intensity and what type of stakeholder participation is needed in relation to problem types addressed in research projects (including non-wicked ones).
